# Electroencephalography microstates imbalance across the spectrum of early psychosis, autism, and mood disorders

**DOI:** 10.1192/j.eurpsy.2023.2414

**Published:** 2023-05-29

**Authors:** Anton Iftimovici, Angela Marchi, Victor Férat, Estelle Pruvost-Robieux, Eléonore Guinard, Valentine Morin, Yannis Elandaloussi, Arnaud D’Halluin, Marie-Odile Krebs, Boris Chaumette, Martine Gavaret

**Affiliations:** 1 Université Paris Cité, Institute of Psychiatry and Neuroscience of Paris (IPNP), INSERM U1266, Paris, Institutde psychiatrie, CNRS GDR 3557, France; 2 NeuroSpin, Atomic Energy Commission, Gif-sur Yvette, France; 3 Pôle PEPIT, GHU Paris Psychiatrie et Neurosciences, Paris, France; 4 APHM, Timone Hospital, Epileptology and Cerebral Rhythmology, Marseille, France; 5 Functional Brain Mapping Laboratory, University of Geneva, Geneva, Switzerland; 6 Neurophysiology and Epileptology department, GHU Paris Psychiatrie et Neurosciences, Paris, France; 7 Department of Psychiatry, McGill University, Montréal, QC, Canada

**Keywords:** Electroencephalography, EEG microstates, psychosis, mood disorders, autism spectrul disorder, transdiagnostic approaches

## Abstract

**Background:**

Electroencephalography (EEG) microstates translate resting-state temporal dynamics of neuronal networks throughout the brain and could constitute possible markers of psychiatric disorders. We tested the hypothesis of an increased imbalance between a predominant self-referential mode (microstate C) and a decreased attentional mode (microstate D) in psychosis, mood, and autism spectrum disorders.

**Methods:**

We retrospectively included 135 subjects from an early psychosis outpatient unit, with available eyes-closed resting-state 19 electrodes EEG. Individual-level then group-level modified *K*-means clustering in controls provided four microstate maps that were then backfitted to all groups. Differences between microstate parameters (occurrence, coverage, and mean duration) were computed between controls and each group, and between disease groups.

**Results:**

Microstate class D parameters were systematically decreased in disease groups compared with controls, with an effect size increasing along the psychosis spectrum, but also in autism. There was no difference in class C. C/D ratios of mean duration were increased only in SCZ compared with controls.

**Conclusions:**

The decrease in microstate class D may be a marker of stage of psychosis, but it is not specific to it and may rather reflect a shared dimension along the schizophrenia-autism spectrum. C/D microstate imbalance may be more specific to schizophrenia.

## Introduction

Psychosis affects approximately 3% of the population, usually in adolescence or early adulthood [1], with major functional, social, and occupational impacts. However, the recent deployment of early intervention has allowed us to revise our view of schizophrenia (SCZ), which now appears as the chronic stage of a disorder that could be prevented if appropriate care were provided at earlier stages [2–4]. Although it is possible to identify subjects with attenuated psychotic symptoms (subthreshold hallucinations or delusions), known as “ultra-high risk” for psychosis (UHR), it remains very difficult to predict clinical outcomes. Approximately a quarter to one-third of UHR will have a first episode of psychosis (FEP), mostly during the first year [5, 6]. Adding to this complexity, the UHR and FEP stages may be preceded by nonspecific symptoms of anxiety and major depressive disorder (MDD) [7]. Psychosis is frequently comorbid with autism spectrum disorders (ASD) [8] and progression can lead not only to SCZ but also to mood disorders [9]. Such a phenotypic overlap is explained by shared environmental stressors (e.g. cannabis, social stress) [10], genetic [11, 12], and structural brain similarities across diseases [13]. In light of this clinical and biological continuum, a transdiagnostic approach is required to identify diagnostic and prognostic biomarkers.

Electroencephalography (EEG) is a simple, accessible, and inexpensive tool already widely used in routine psychiatric practice to rule out possible neurological comorbidities. From a frequency point of view, abnormalities of neural oscillations or evoked potentials in EEG are well known in psychosis and particularly during late adolescence [14]. But it is also possible to study the resting-state temporal dynamics of neuronal networks throughout the brain, from a spatial perspective [15]. At rest, brain activity alternates very rapidly, around every 100 ms, between states of unstable equilibrium, called EEG microstates, and characterized by a particular polarization of the electric potential field across the whole brain [16]. In the global neuronal workspace framework [17], these microstates may correspond to the spatial organization of particular modes of information processing [16]. Of the four microstates classically described (A, B, C, and D), two are especially relevant for psychosis. Microstate C has been correlated with positive BOLD activations in the resting-state networks involved in the integration of interoceptive information associated with emotional salience, and it may reflect a predominantly self-referential mode [18]. Microstate D was correlated with negative BOLD signals in resting-state networks involved in attentional aspects, such as the detection of relevant behavioral stimuli and the ability to switch and redirect attention [18].

It has been postulated that psychosis would be characterized by an imbalance between a predominant self-referential mode (microstate C) and a decreased attentional mode (microstate D), reflecting a disconnection between mental states on the one hand, and information received from the environment on the other. Indeed, an increased ratio of microstate C over D has already been reported in SCZ [19], while antipsychotic treatment seems to increase the duration of class D [20, 21]. At earlier stages, microstate analysis provided more conflicting results. UHR subjects who subsequently have a first psychotic episode appear to have less class D in general, but no significant results are found for class C [22]. Relatives of subjects with SCZ have more class C and less class D [23], which is also the case for subjects with 22q11.2 deletion, who are at a very high genetic risk of psychosis (more than 40% of them will develop a psychotic disorder) [24]. Conversely, a study comparing treated and untreated FEP did not find significant differences between classes C and D [25]. Moreover, while anomalies in the sequence of these microstates have been suggested as a possible endophenotype of SCZ spectrum disorders, there is not, to the best of our knowledge, any transdiagnostic study addressing the specificity of such a biomarker, across the spectrum of psychosis and compared to autism and mood spectrum disorders. Notably, a class D decrease has also been proposed to be a biomarker for depression [26–28], whereas the distribution of class C and D in autism remains equivocal, with a trend of less class C compared to controls [29–32].

Here, we present the first transdiagnostic study of the imbalance between microstates C and D in psychosis spectrum disorders (ultra-high-risk [UHR] of psychosis, FEP, recent-onset SCZ), mood disorders (MDD and bipolar disorder [BIP]), and ASD, in comparison with controls without a psychiatric disorder, using routine clinical EEG setting.

## Methods

### Participants

This retrospective study did not modify the usual medical practices, allowing the recruitment of patients whose EEG was recorded as part of routine and systematic clinical evaluation. All patients and controls provided informed consent following the regulation authorities, under the promotion of the DRCI (Direction Recherche Clinique Innovation) of the GHU Paris Psychiatry and Neurosciences. We retrospectively screened all subjects assessed between 2018 and 2020 at the early psychosis outpatient unit (C’JAAD), who underwent at the same time an EEG recording in the neurophysiology department of the GHU Paris Psychiatry and Neurosciences. The inclusion criteria of the study were as follows: (1) a diagnosis of ultra-high risk diagnosis of psychosis, according to the CAARMS scale [33] in its French-translated version [34], or of first episode psychosis, SCZ, MDD, BIP, or ASD, defined according to the DSM-5 nomenclature and the positive Autism Diagnostic Observation and Autism Diagnostic Interview-Revised scales for autism diagnosis [35]; and (2) to be untreated (antipsychotics, antidepressants, or benzodiazepines), or to have minimal treatment, introduced only recently (less than 2 weeks). The exclusion criteria of the study were as follows: a severe underlying neurological disease (epilepsy, brain injury, etc.) with abnormal brain MRI or abnormal EEG. Drug use (especially cannabis) was not an exclusion criterion, and could not be evaluated because of the retrospective nature of the study. Control subjects were also included retrospectively among patients assessed in the neurophysiology department for migraine, who presented a normal EEG and strictly no other neurological or psychiatric sign or symptom after specialist evaluation.

### EEG recording and preprocessing

Routine clinical EEGs had a mean duration of 20 ± 3 min. They were performed at rest in a half-seated position, and included rest sequences with eyes closed, and eyes open, followed by stimulation procedures (hyperpnea and then intermittent photic stimulation), all of which were annotated. Only sequences at rest with eyes closed and before hyperpnea were kept for analysis. The EEG setup was 19 electrodes, following the international 10–20 system [36]. Sampling rate was 256 Hz. Raw EEG recordings were stored in BIDS format following international recommendations [37]. Preprocessing was done using the MNE EEG software on Python [38], following the OHBM COBIDAS MEEG good practice recommendations [39]. Notably, a bandpass filter between 0.5 and 40 Hz was applied before the segmentation, in order to avoid edge effects, and segmentation with the elimination of sequences annotated other than “eyes closed” was done before re-referencing to the mean, to avoid spatial extension of artifacts [39]. Next, each trace was visually reanalyzed to ensure that it was indeed an alpha-dominant, resting rhythm without any residual artifact. Finally, a last quality check of the EEGs was done for each EEG separately, using the spectral power density graph, to also ensure that frequencies between 8 and 13 Hz were predominant. Only one artifacted channel per EEG was tolerated, and in this case, interpolated. EEGs with more than one artifacted channel were excluded from the analysis.

### Microstate analysis

Microstate analysis was done using the Pycrostates package developed by Victor Férat (github.com/vferat/pycrostates) [40]. Global field power (GFP) was determined for each participant. Only EEG topographies at GFP peaks were retained to determine microstates’ topographies, through a modified K-means clustering. The silhouette score provided an optimal number of clusters of four, which allowed comparison with the literature (Supplementary Figure S1). We checked the comparability between clusters of each group by applying a two-step clustering: at the subject level, we computed individual topographies from GFP peaks; then, at the group level, we concatenated all individual topographies from the same group into a single dataset for a second round of clustering. We then computed the spatial correlation between each group’s maps and the control maps. To allow comparison across groups and obtain reference topographies independent of psychiatric pathologies, we backfitted the control maps on the whole EEG recordings of the disease groups. Temporal smoothing (82 ms full window size) was applied to ensure that periods of inter-peak noise, of low GFP, did not interrupt the sequences of quasi-stable segments [41]. For each subject, three parameters were computed for each class: frequency of occurrence (“occurrence”), temporal coverage (“coverage”) and mean duration. Occurrence is the average number of times a given microstate occurs per second. Coverage (in %) is the percentage of total analysis time spent in a given microstate. Mean duration (in ms) is the average time during which a given microstate was present in an uninterrupted manner (after temporal smoothing).

### Statistical analysis

Comparisons of the three microstate parameters (duration, coverage, occurrence) were done for class C, class D, and the ratio of class C over D, between each disease group and controls. We tested the hypothesis that each disease group significantly differed from the controls for values of parameters of class C, class D, and ratios of class C over D. To allow comparison between groups, we used Bayesian estimation with the BEST code in Python [42, 43]. A prior t-distribution was assumed before a Markov chain Monte Carlo algorithm was applied to estimate the posterior distribution. This provided posterior estimations of the means, standard deviations, and effect sizes of the difference between each group and the controls. The measure of statistical certainty was the posterior probability for the effect sizes to be outside of the region of practical equivalence (ROPE), defined for an effect size as the interval between −0.1 and 0.1 [42], with a threshold set at 95%. Differences in class C, D, and C/D ratios among all disease groups, and for each of the three parameters separately, were computed with a one-way ANOVA, adjusted for age and sex. Applicability of ANOVA was verified: (i) the groups were independent; (ii) each sample came from a normally distributed population, which we tested by comparing each group against a normal distribution; and iii) homoscedasticity was true, with equal standard deviations amongst disease groups, based on the Levene test.

## Results

### Characteristics of the dataset

**Figure 1.**
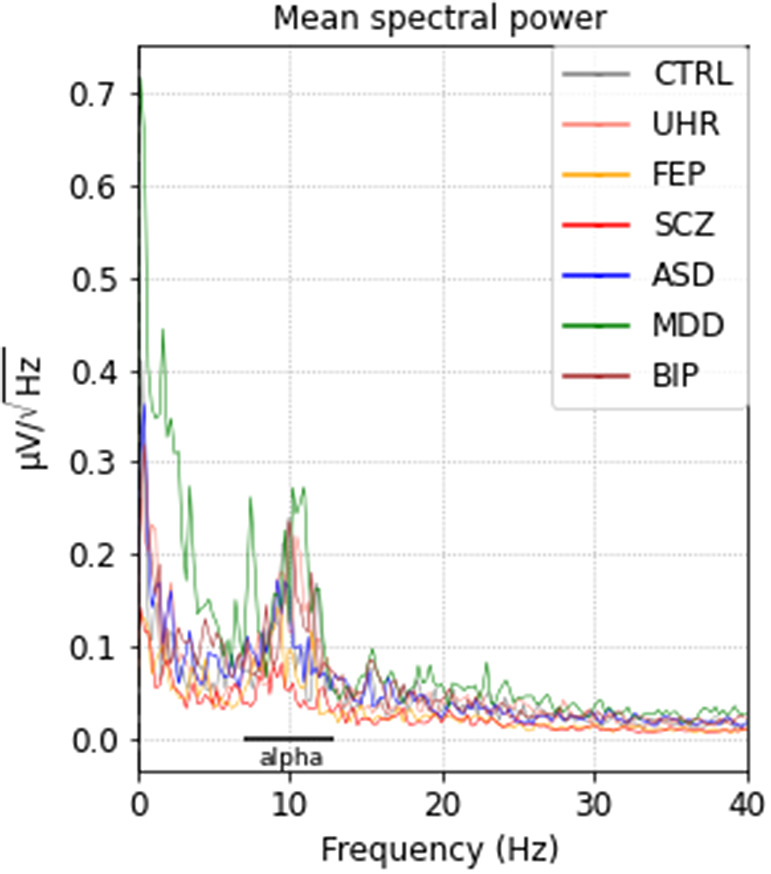
Mean spectral power densities for each group after preprocessing. ASD, autism spectrum disorder; BIP, bipolar disorder; CTRL, controls; FEP, first-episode psychosis; MDD, major depressive disorder; SCZ, schizophrenia; UHR, ultra-high-risk.

We screened a total of 195 patients with psychiatric diseases and excluded 60 patients who had an EEG of poor quality. Along the remaining 135 patients, 11 control subjects were also included. Population characteristics are summarized in Table 1. After preprocessing the closed-eyes resting-state recordings, all groups showed an equivalent predominance of alpha-band frequency, between 8 and 13 Hz (Figure 1).

**Table 1.** Population.

	CTRL	UHR	FEP	SCZ	ASD	MDD	BIP	F/χ2	*p*
Number of subjects	11	22	25	34	21	19	14	–	–
Age (mean ± std)	31 ± 13	20.7 ± 3.6	20.7 ± 4.5	21.8 ± 3.8	18.5 ± 3.0	19.5 ± 2.9	19.5 ± 3.6	2.55	**0.031**
Sex (F/M)	5/6	8/14	12/13	11/23	7/14	9/10	7/7	1.79	0.773
EEG duration (min)	3.1 ± 4.4	2.4 ± 0.6	2.5 ± 0.8	2.4 ± 0.8	2.1 ± 1.0	2.3 ± 0.7	2.6 ± 1.0	0.84	0.524

*Note*: ANOVA and χ2 were computed including all groups except controls.

Abbreviations: ASD, autism spectrum disorder; BIP, bipolar disorder; CTRL, controls; FEP, first-episode psychosis; MDD, major depressive disorder; SCZ, schizophrenia; UHR, ultra-high-risk.

Bolded value stands for Statistically significant value (*p* < 0.05).

### Microstate topographies

**Figure 2.**
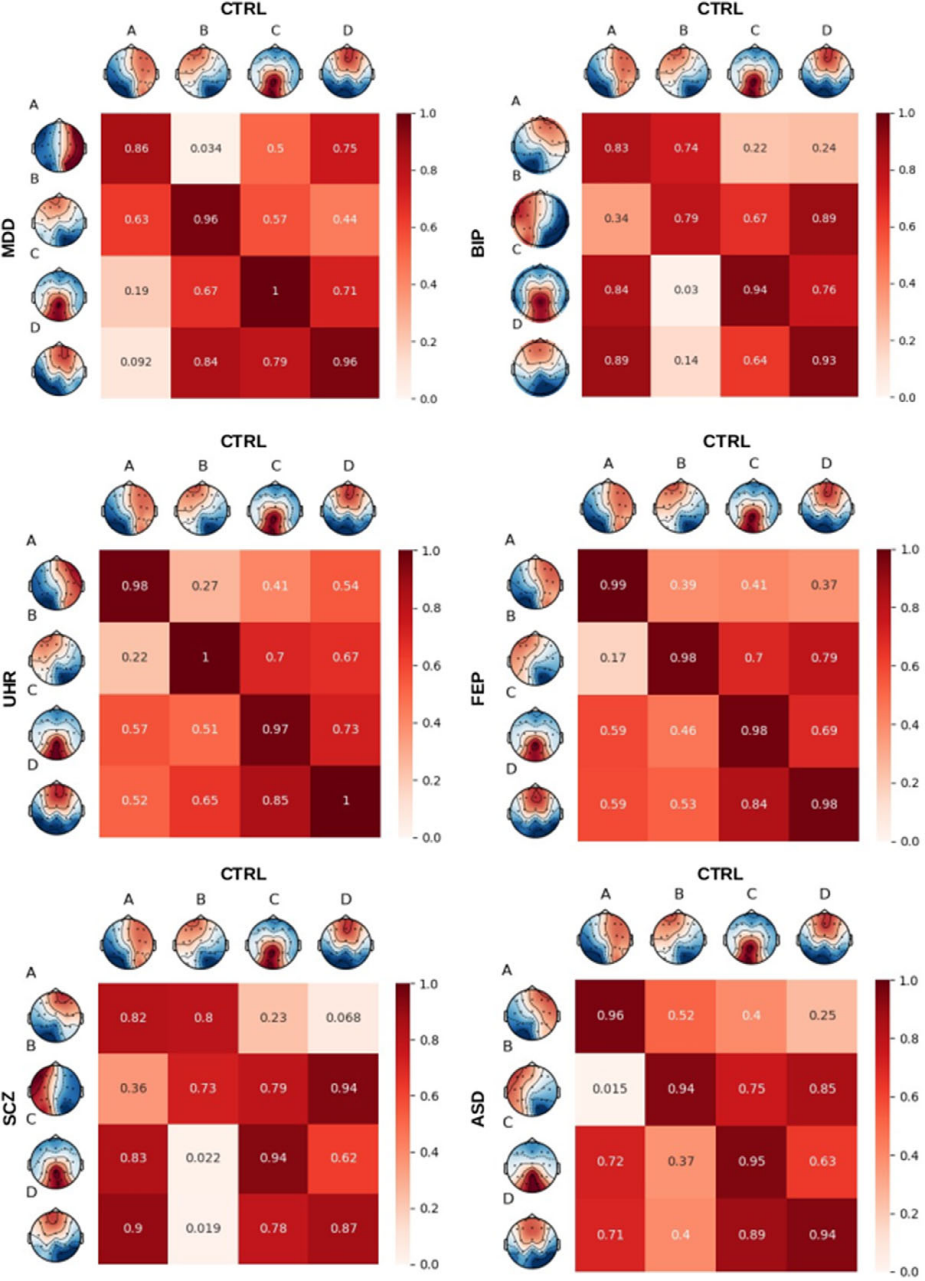
Spatial correlations between the four clusters of each disease group and the controls.

The modified *K*-means clustering in four maps found topographies A, B, C, and D, similar to what has been reported in the literature. The average absolute spatial correlation between the class maps of controls and each of the groups was high: 0.91 for class A, 0.90 for class B, 0.96 for class C, and 0.95 for class D (Figure 2). The distribution of maps was most similar between major depression disorder, UHR, FEP, ASD, and controls. Less stable maps were found in the BIP and SCZ groups. In this case, despite a relatively high inter-group spatial correlation between the same classes, we observed in some cases higher correlations between different classes. This reflects less stability and reliability of the respective maps.

### Comparison of class C, class D, and class C/class D ratios

Regarding class C, there was no significant difference between groups and controls, with not any significant effect-size for microstate parameters of class C.

**Figure 3.**
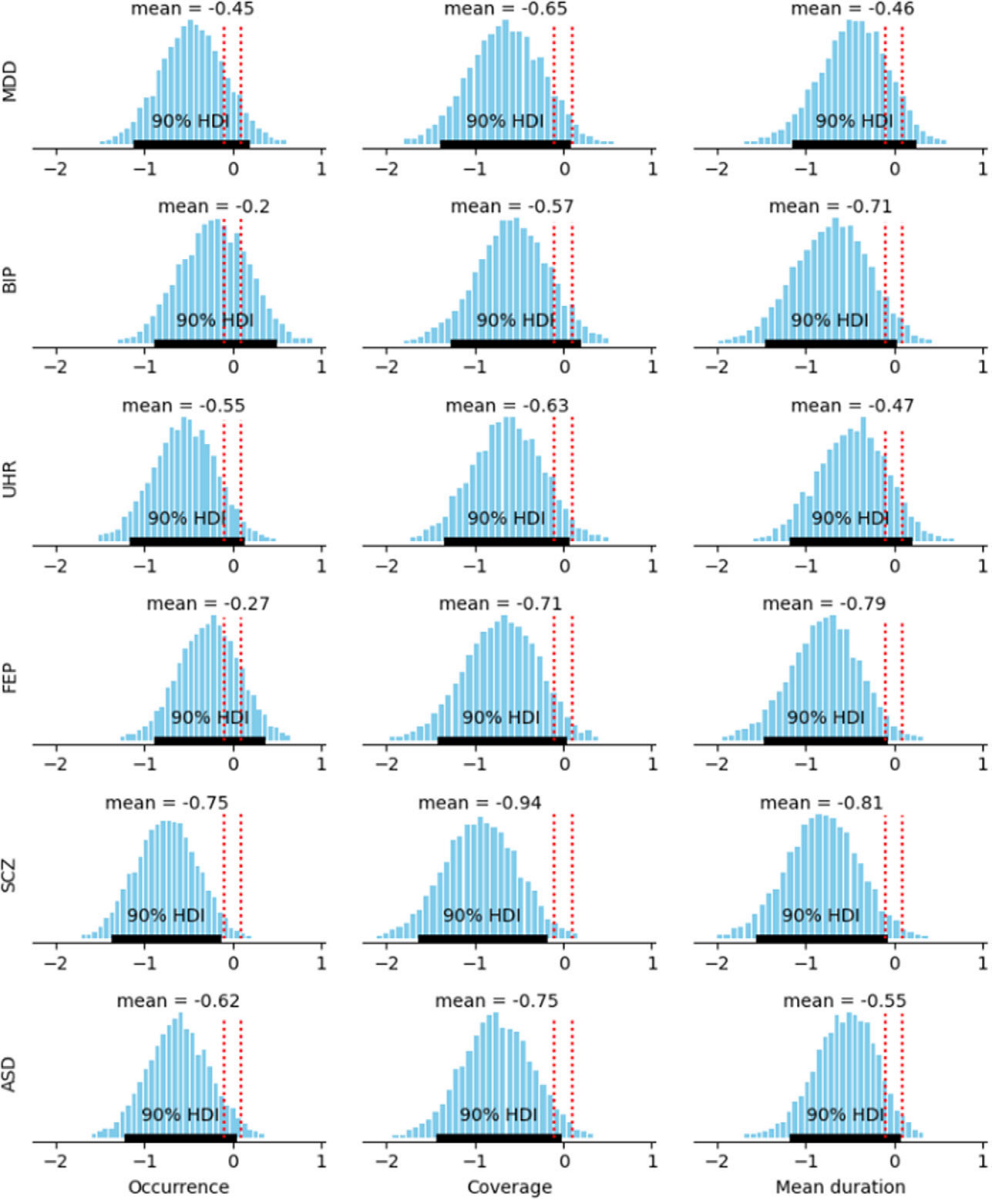
Posterior estimation of the effect-size of the difference in microstate D parameters (occurrence, coverage, mean duration) between each disease group and the controls. The red dotted interval represents the region of practical equivalence. HDI: 90% highest density interval of the posterior estimation.

Regarding class D, there was an overall decrease in all groups compared to controls. For occurrence, the effect-size of the difference with controls was significant for SCZ and ASD. For coverage, the effect-size of the difference with controls was significant for FEP, SCZ, and ASD. For the mean duration, the effect-size of the difference with controls was significant for BIP, FEP, and SCZ (Figure 3, Table 2).

**Table 2.** Comparisons of the ratios of class C over class D for each microstate parameter (occurrence, coverage, mean duration), between each disease group and the controls.

Microstate parameter	Group minus Controls	Class C		Class D		Ratio Class C/Class D	
		Effect-size (mean)	Posterior probability (%)	Effect-size (mean)	Posterior probability (%)	Effect-size (mean)	Posterior probability (%)
Occurrence	MDD	0.01	78	−0.45	89	0.25	83
	BIP	0.56	93	−0.20	82	0.54	91
	UHR	0.13	78	−0.55	93	0.40	88
	FEP	0.42	89	−0.27	83	0.42	90
	SCZ	0.28	83	−0.75	98	0.57	94
	ASD	0.28	84	−0.62	95	0.56	93
Coverage	MDD	0.08	78	−0.65	94	0.28	84
	BIP	0.30	85	−0.57	93	0.56	92
	UHR	0.18	80	−0.63	94	0.42	89
	FEP	0.24	83	−0.71	96	0.47	91
	SCZ	0.22	81	−0.94	98	0.47	92
	ASD	0.10	79	−0.75	96	0.40	88
Mean duration	MDD	0.09	78	−0.46	89	0.41	89
	BIP	−0.19	81	−0.71	95	0.49	91
	UHR	0.15	79	−0.47	89	0.44	89
	FEP	−0.18	80	−0.79	97	0.59	94
	SCZ	0.16	79	−0.81	96	0.63	95
	ASD	−0.02	78	−0.55	94	0.38	87

*Note: The posterior probability is the probability for the effect size of the difference to be outside of the region of practical equivalence, between −0.1 and 0.1.*

Abbreviations: ASD, autism spectrum disorder; BIP, bipolar disorder; FEP, first-episode psychosis; MDD, major depressive disorder; SCZ, schizophrenia; UHR, ultra-high-risk.

Regarding the class C / class D ratio, only the effect-size of the difference between SCZ and controls was significant (Table 2).

The ANOVA analysis found no significant difference for any microstate parameter among disease groups (Supplementary Figure S2 and Supplementary Table S1). However, regarding class B, the effect-size of the difference with controls was also significantly positive for occurrence (in BIP, FEP, SCZ, and ASD) and for coverage (in FEP, SCZ, ASD) (Supplementary Figure S3 and Supplementary Table S2).

## Discussion

In this retrospective study, we presented a transnosographic EEG microstate analysis across psychosis spectrum disorders, mood disorders, and ASD based on routine recordings. We investigated whether the hypothesized imbalance between an increased microstate C and a decreased microstate D was characteristic of any specific stage of psychosis, from UHR to FEP and SCZ, or whether this was also found in MDD and BIP, as well as ASD. To this end, we applied a spatial modified-k-means clustering to the EEG recordings, during resting-state with eyes closed, of seven independent groups (the six disease groups and a control group). First, we obtained microstate topographies independent of any psychiatric disorder by building a clustering in the control group. We observed the same four microstate topographies as those reported in the literature, especially microstate C, thought to be related to interoception and a self-referential mode, and microstate D, which may involve externally driven aspects of attention [18]. An increase in the ratio of microstates C over D could therefore reflect the imbalance in information processing previously reported in subjects with psychosis. Delusion may stem from an unwavering adherence to an internal idea despite contradictory external information, a hypothesis in line with the loss of context described by Jasperian phenomenology as one of the roots of delusion [44].

The high spatial correlation between maps built independently in each disease group and those from the controls allowed us to apply the control topographies to all disease groups, and compute three parameters (occurrence, coverage, and mean duration) of each microstate in each disorder. We could then assess for each parameter their ratios of microstate C over microstate D and compare them with controls. Microstate class C parameters were not different between groups and controls. This is in line with a recent report of no increase in microstate C in early psychosis [22]. Conversely, microstate class D parameters were systematically decreased in disease groups compared with controls. This was especially significant for FEP (coverage and mean duration), SCZ (occurrence, coverage, and mean duration), and ASD (occurrence and coverage). Interestingly, the effect size of the difference with controls increased along the psychosis spectrum for class D coverage (UHR: –0.63; FEP: –0.71; SCZ: −0.94) and mean duration (UHR: –0.47; FEP: -0.79; SCZ: −0.81). These results suggest that class D parameters may be markers of stage along the psychosis spectrum. However, since ASD also showed significant decreases in class D occurrence and coverage, class D parameter decrease may not be specific to psychosis and may rather reflect a shared dimension on the SCZ-autism spectrum. This is not surprising as class D is believed to be a broad proxy measure for external attention, and it further illustrates at the EEG microstate level the continuum between psychosis and ASDs that is well described from genetics to neuroimaging [12, 13]. Nevertheless, when directly looking at the imbalance between class C and class D, it only differs significantly from controls for the mean duration in SCZ subjects, while for each parameter (occurrence, coverage, and mean duration), there is an increase along the psychosis spectrum from UHR, to FEP, to SCZ. This increase of imbalance during the emergence of psychosis coincides with the physiological increase in C and decrease in D that normally occur in healthy subjects between 16 and 25 years [45], suggesting a level of continuity between health and disease. These results further align with the excitatory/inhibitory imbalances and loss in oscillatory power that have been reported to occur in the same time frame of late adolescence and early adulthood, and which have been suggested to underlie psychotic risk [14]. Overall, our results tentatively suggest that the imbalance of class C over class D may be more specific to SCZ than the class D variations by themselves.

Several considerations need to be made. First, when comparing values of all parameters between diseases, no significant difference was found. This could indicate that while there may be a significant difference in certain class D values or C/D ratios of microstate parameters between controls and patients, this difference is not sufficient to discriminate between disease groups, and therefore lacks disease specificity. Furthermore, significant increases of class B in BIP (occurrence), FEP, SCZ, and ASD (occurrence and coverage) show that microstate B, which may relate to visual processing [16], may also be relevant transnosographically. Although this observation is intriguing, it remains tentative since the topography of class B in our dataset may be less stable and at times, correlates with the topography of class D (in BIP and SCZ subjects). In addition to the limited size of the samples, the absence of difference between groups may be explained by the heterogeneity behind each disease category: some UHR will convert to psychosis, while others will not, and it has been shown that microstate D is decreased in converters compared to non-converters [22]. Similarly, subjects experiencing a first episode of psychosis will not all develop SCZ, and around 40% will recover (for more than 2 years) [46, 47], with important variability and very different long-term functional outcomes [48].

Second, our study was carried out in routine care and therefore presents the advantage of estimating the performance of microstate analysis in a heterogeneous population as seen in daily practice, with routine 19 electrodes EEG. The disadvantage is that 19 electrodes EEG are also less reliable and noisier than higher density ones for microstate analysis [49]. Transdiagnostic studies with high-resolution EEG are therefore required, with longer eye-closed recordings specifically dedicated to microstate analysis (e.g. 5 min), as they provide more robust results [23]. Nevertheless, the spatial correlation that we observed for maps across groups suggests a level of stability even at this low resolution, provided the raw EEG data is clean. Yet, the retrospective nature of our study did not allow for more specific quantitative phenotyping that would have provided information on the relationship between microstates and clinical dimensions, or precise medication intake.

Third, a specificity of our study is to have chosen to construct the microstate prototypes from healthy subjects only. The microstate topographies thus characterized translate a normal variation and are independent of the disease groups to which they were applied. This allowed us to amplify a signal possibly associated with the disease, which would otherwise have been attenuated by strategies that construct these clusters by combining all groups.

Several methodological limits need also to be mentioned. Although the overall cohort is large, the sample size of each group is relatively small. For this reason, data analysis was done with Bayesian estimation, which has been shown to be more robust than *t*-tests for small sample sizes [42]. The optimal number of clusters was determined using a silhouette score that identified two and four clusters as possible solutions, but we chose the latter to maintain comparability both with the literature and across multiple groups. Moreover, there is not, to date, a consensus regarding the criterion for finding the optimal number of maps, and most of the clustering is driven by the underlying hypothesis: we chose a fixed number of clusters because we wanted to compare groups, assuming there is no qualitative difference between disorders; but an alternative strategy could have been to seek for maps specific to one disorder by allowing the optimal number of clusters to vary from one group to another [16]. Finally, while age and sex were included as covariates in the ANOVA model for differences among disease groups, we could not correct for age in the pairwise comparisons against controls, because age and status were strongly associated (as all controls were significantly older than all disease groups). This is a limit of the retrospective design, and a prospective one will allow to include controls matched on age and sex.

In conclusion, we reported here the first transdiagnostic study of EEG microstates across the spectrum of early psychosis, autism, and mood disorders in routine clinical practice. Our results suggest that class D decreases may be markers of stage across psychosis, but they do not seem to be specific to it and may also reflect a shared dimension on the SCZ-autism spectrum. On the other hand, we also tentatively suggest that the C/D microstate ratio imbalance, which is postulated to reflect core contextualization deficits of psychosis, may be more specific to SCZ. At this stage, EEG microstates are not sufficient to differentiate between different groups of diseases.

## Supplementary material

For supplementary material accompanying this paper visit http://doi.org/10.1192/j.eurpsy.2023.2414. click here to view supplementary material
