# Supplementary material for: Electroencephalography microstates imbalance across the spectrum of early psychosis, autism, and mood disorders
Source: Eur Psychiatry. 2023 May 29;66(1):e41. doi: 10.1192/j.eurpsy.2023.2414 (PMC10305759; doi:10.1192/j.eurpsy.2023.2414)
Supplement: Supplementary file 1 [file S0924933823024148sup001.pdf]

SUPPLEMENTARY DATA

Supplementary Figure 1 : Silhouette score

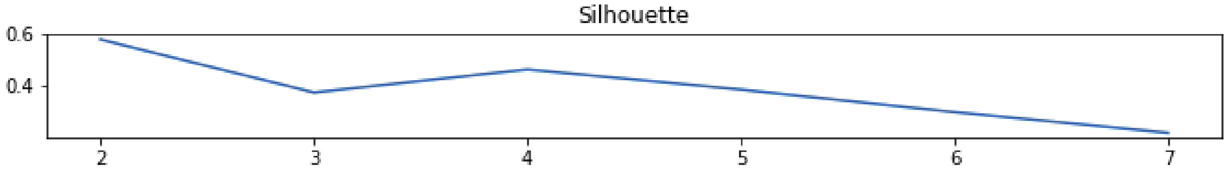

Supplementary Figure 2 : Distribution of each microstate parameter across each group and for each microstate class

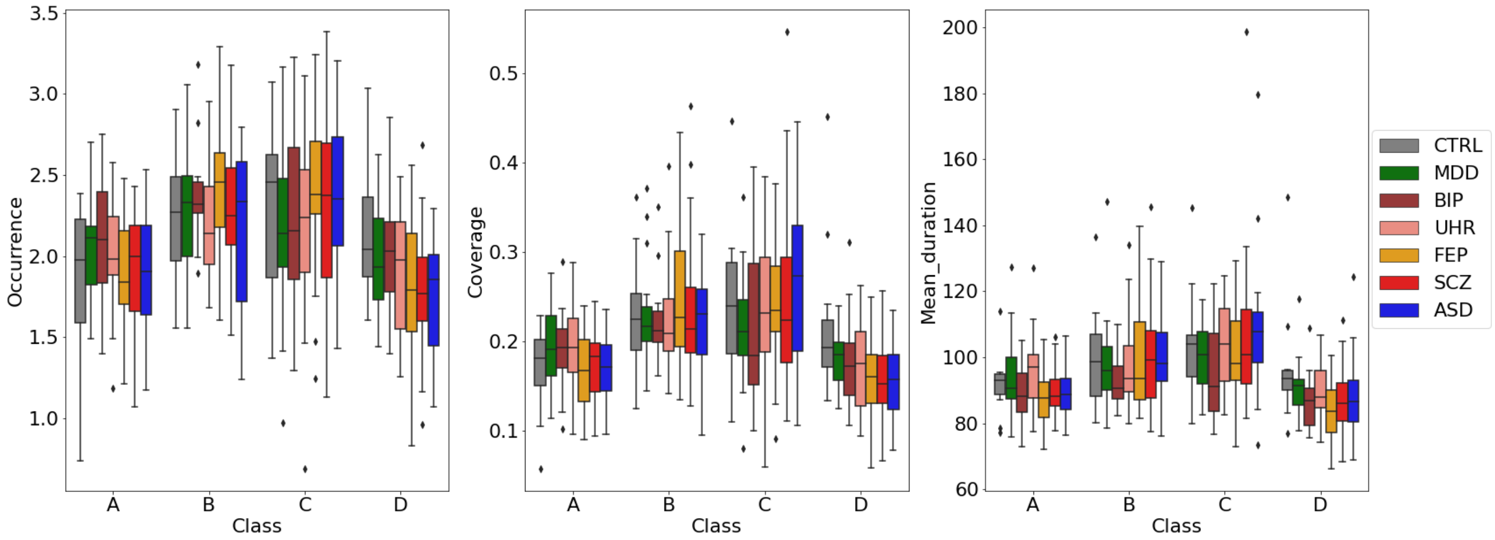

Supplementary Figure 3: Posterior estimation of the effect-size of the difference in microstate B parameters (occurrence, coverage, mean duration) between each disease group and the controls. The red dotted interval represents the region of practical equivalence. HDI: 90% highest density interval of the posterior estimation.

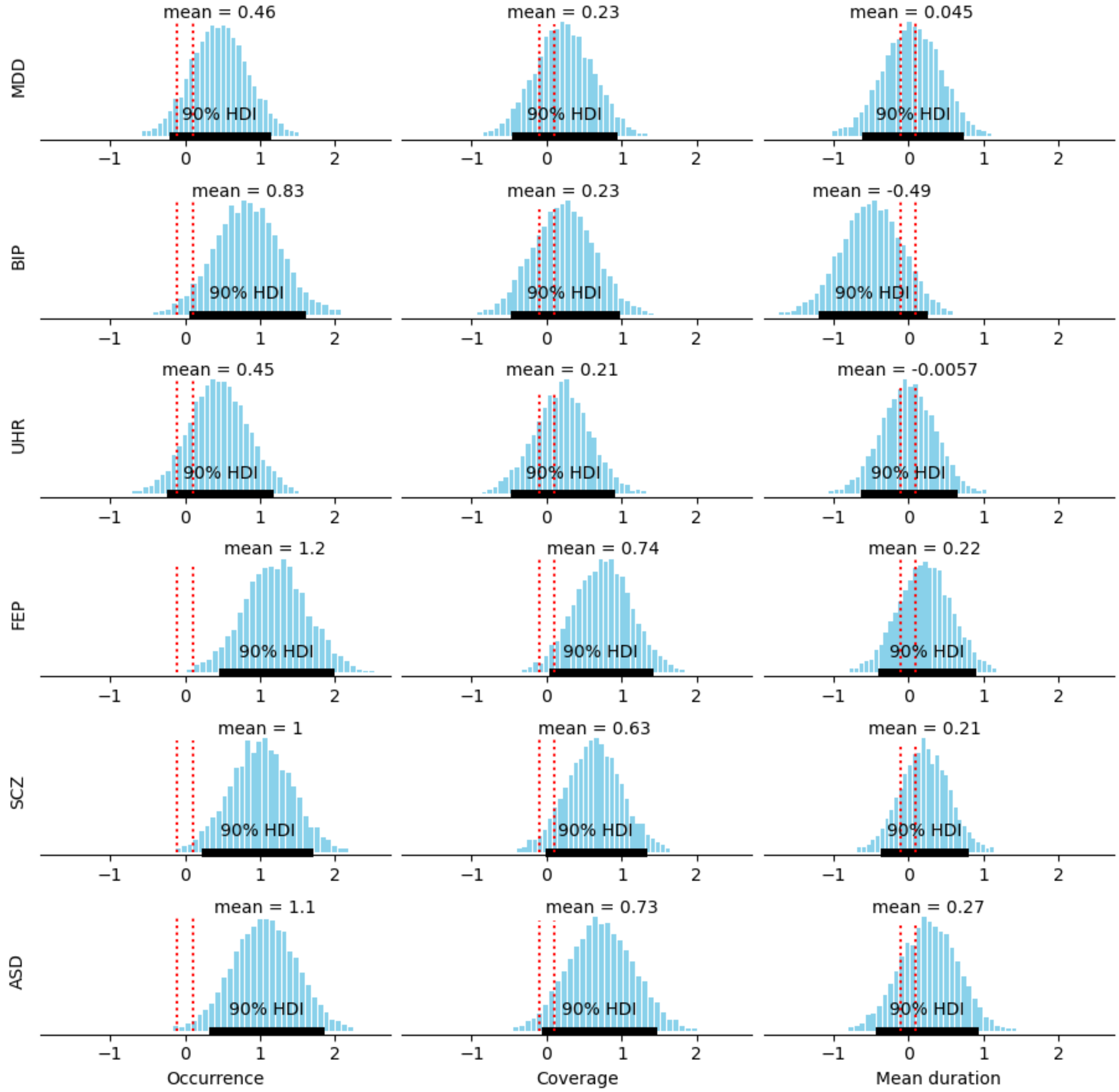

Supplementary Table 1 : Mean and standard deviations of each microstate parameter (occurrence, coverage, mean duration) in each group and each microstate class. ANOVA was computed on all disease groups (excluding controls) to test for intra-disease differences.

Microstate parameter	Class	CTRL	MDD	BIP	UHR	FEP	SCZ	ASD	F-statistic	p-value
**Occurrence (/s)**	**A**	1.7 ± 0.6	2.0 ± 0.3	2.1 ± 0.4	2.0 ± 0.3	1.9 ± 0.3	1.9 ± 0.3	1.9 ± 0.4	1.18	0.321
	**B**	2.1 ± 0.1	2.3 ± 0.4	2.4 ± 0.3	2.2 ± 0.3	2.4 ± 0.4	2.3 ± 0.4	2.2 ± 0.5	1.19	0.312
	**C**	2.1 ± 0.7	2.1 ± 0.5	2.3 ± 0.6	2.2 ± 0.5	2.4 ± 0.5	2.3 ± 0.5	2.4 ± 0.5	0.84	0.521
	**D**	2.3 ± 0.6	2.0 ± 0.3	2.0 ± 0.4	1.9 ± 0.4	1.8 ± 0.4	1.8 ± 0.4	1.7 ± 0.4	1.91	0.096
	**E**	1.6 ± 0.6	2.0 ± 0.3	2.0 ± 0.5	1.9 ± 0.3	2.0 ± 0.3	2.0 ± 0.3	1.9 ± 0.5	0.63	0.676
	**Ratio C / D**	1.1 ± 0.4	1.1 ± 0.3	1.2 ± 0.5	1.2 ± 0.5	1.4 ± 0.6	1.3 ± 0.5	1.5 ± 0.5	1.94	0.091
**Coverage (%)**	**A**	16 ± 6	20 ± 5	19 ± 5	19 ± 5	17 ± 4	17 ± 4	17 ± 4	1.98	0.086
	**B**	22 ± 8	23 ± 6	22 ± 5	22 ± 6	25 ± 8	23 ± 7	22 ± 6	0.58	0.710
	**C**	23 ± 10	22 ± 6	22 ± 9	23 ± 8	25 ± 7	24 ± 10	27 ± 9	0.90	0.480
	**D**	21 ± 10	18 ± 3	18 ± 6	17 ± 5	16 ± 4	16 ± 4	16 ± 5	1.65	0.150
	**E**	14 ± 5	18 ± 4	18 ± 7	18 ± 6	18 ± 4	20 ± 6	19 ± 7	0.31	0.903
	**Ratio C / D**	1.3 ± 0.6	1.3 ± 0.4	1.4 ± 0.8	1.5 ± 0.9	1.8 ± 1.0	1.8 ± 1.4	2.0 ± 1.2	1.52	0.186
**Mean duration (ms)**	**A**	85 ± 25	95 ± 12	89 ± 9	96 ± 11	88 ± 8	89 ± 7	89 ± 9	2.86	**0.018**
	**B**	93 ± 28	99 ± 14	93 ± 8	98 ± 13	100 ± 16	100 ± 16	99 ± 12	0.52	0.754
	**C**	97 ± 29	100 ± 10	95 ± 15	104 ± 14	101 ± 13	105 ± 21	109 ± 21	1.40	0.226
	**D**	91 ± 28	91 ± 8	87 ± 7	91 ± 8	84 ± 9	86 ± 9	88 ± 13	1.57	0.172
	**E**	78 ± 23	91 ± 12	88 ± 10	92 ± 15	90 ± 9	94 ± 19	95 ± 17	0.66	0.648
	**Ratio C / D**	1.1 ± 0.3	1.1 ± 0.2	1.1 ± 0.2	1.2 ± 0.2	1.2 ± 0.3	1.3 ± 0.4	1.3 ± 0.4	1.03	0.398

Supplementary Table 2: Comparisons of class A and class B for each microstate parameter (occurrence, coverage, mean duration), between each disease group and the controls. MDD: major depressive disorder. BIP: bipolar disorder. UHR: ultra-high-risk. FEP: first-episode psychosis. SCZ: schizophrenia. ASD: autism spectrum disorder. The posterior probabilities is the probability for the effect size of the difference to be outside of the region of practical equivalence, between -0.1 and 0.1.

Microstate parameter	Group minus Controls	Class A		Class B	
		Effect-size (mean)	Posterior probability	Effect-size (mean)	Posterior probability
**Occurrence**	**MDD**	0.41	88%	0.47	90%
	**BIP**	0.48	90%	0.85	97%
	**UHR**	0.36	87%	0.45	89%
	**FEP**	0.25	83%	1.19	99%
	**SCZ**	0.30	85%	1.00	99%
	**ASD**	0.26	84%	1.06	99%
**Coverage**	**MDD**	0.49	91%	0.23	83%
	**BIP**	0.35	87%	0.21	84%
	**UHR**	0.45	89%	0.22	82%
	**FEP**	0.02	79%	0.74	97%
	**SCZ**	0.27	83%	0.63	95%
	**ASD**	0.25	83%	0.73	96%
**Mean duration**	**MDD**	0.45	90%	0.05	79%
	**BIP**	-0.12	81%	-0.49	90%
	**UHR**	0.46	90%	-0.02	78%
	**FEP**	-0.44	90%	0.22	82%
	**SCZ**	0.04	74%	0.20	80%
	**ASD**	0.1	79%	0.27	84%
